
JOURNAL INFORMATION
==============================
NLM Title Abbreviation: JBRA Assist Reprod
Journal ID: JBRA Assist Reprod
NLM Journal ID: 101684552
Journal ID: jbra

JBRA Assisted Reproduction

ISSN: 1517-5693
EISSN: 1518-0557
Publisher: Brazilian Society of Assisted Reproduction

ARTICLE INFORMATION
==============================
PMCID: PMC6501738
PMID: 31038295
DOI: 10.5935/1518-0557.20190025
Article version: 1
Subjects: Oral Presentations

Abstracts of the 14th Red Lara Taller General, Mérida, Mexico, 06-9 March 2019

Publication date: 2019 Apr-Jun
Print publication date: 2019 Apr-Jun
Volume: 23
Issue: 2
First page: 179
Last page: 181
License: This is an Open Access article distributed under the terms of the Creative Commons Attribution License, which permits unrestricted use, distribution, and reproduction in any medium, provided the original work is properly cited.
License URL: https://creativecommons.org/licenses/by/4.0/

==============================


O-01. KIDscore™ D5 algorithm as an additional tool to morphologic assessment and PGT-A for embryo selection: a time-lapse study

Gazzo E 1
Peña F 1
Valdéz F 2
Sessarego S 1
Chung A 1
Bonomini C 1
Ascenzo M 1
Velit M 1
Escudero E 1
1 INMATER Fertility Clinic, Lima, Peru.
2 GENOMICS PERÚ, Lima, Peru.

O-02. Associated factors to pregnancy in intrauterine insemination

Vargas Tominaga LA 1
Alarcón F 1
Vargas A 1
Bernal G 1
Medina A 1
Polo Z 1
1 CFGS - Centro de Fertilidad y Ginecología del Sur - Cusco - Peru.

O-03. Blastocyst contractions are strongly related with aneuploidy, lower implantation rates and slower embryo cleavage timing: time lapse study

Gazzo E 1
Peña F 1
Valdéz F 2
Chung A 1
Velit M 1
Ascenzo M 1
Escudero E 1
1 Clínica de Fertilidad INMATER, Lima, Peru.
2 Genomics Peru, Lima, Peru.

O-04. Immature oocyte incidence: Contributing factors and effects on mature sibling oocytes in intracytoplasmic sperm injection cycles

Braga DPAF 1 2
Zanetti BF 1 2
Setti AS 1 2
Iaconelli Jr. A 1 2
Borges Jr. E 1 2
1 Fertility Medical Group, São Paulo, SP - Brazil.
2 Instituto Sapientiae - Centro de Estudos e Pesquisa em Reprodução Humana Assistida, São Paulo, SP - Brazil.

O-05. Serum microRNA profiling for the identification of predictive molecular markers of the response to controlled ovarian stimulation

Borges Jr. E 1 2
Mulato MGF 3
Setti AS 1 2
Iaconelli Jr. A 1 2
Geraldo MV 3
Braga DPAF 1 2
1 Fertility Medical Group, São Paulo, SP - Brazil.
2 Instituto Sapientiae - Centro de Estudos e Pesquisa em Reprodução Assistida São Paulo, SP - Brazil.
3 Departamento de Biologia Estrutural e Funcional, Instituto de Biologia, Universidade Estadual de Campinas - UNICAMP.

O-06. First custom next-generation sequencing infertility panel in Latin America: design and first results

Lorenzi D 1
Fernández C 1
Bilinski M 1
Fabbro M 1
Galain M 1
Menazzi S 1
Miguens M 2
Nicotra Perassi P 1
Fulco MF 2
Kopelman S 2
Fiszbajn G 1
Nodar F 1 2
Papier S 1 2
1 Novagen. Buenos Aires, Argentina.
2 Centro de Estudios en Genética y Reproducción (CEGYR). Buenos Aires, Argentina.

O-07. Obesity and the possibility of conceiving a child during assisted reproduction treatment: An Argentine experience

Sánchez Páez JC 1
Goméz Arreseygor V 1
Zgrablich P 2
1 Laboratorio de Embriología Clínica, Centro de Reproducción Asistida GESTAR.
2 Unidad de Medicina Reproductiva, Centro de Reproducción Asistida GESTAR.

O-08. Regenerative therapy by endometrial mesenchymal stem cells in thin endometrium with repeated implantation failure. A novel strategy

Tersoglio AE 1
Tersoglio S 1
Salatino DR 1
Castro M 1
Gonzalez A 2
Hinojosa M 1
Castellano O 1
1 International Center for Assisted Reproduction, Mendoza, Argentina.
2 Immunology Laboratory, Central Hospital, Mendoza, Argentina.

RED LARA Award

Best Oral Presentation.

14th RED LARA Taller General, Mérida, Mexico - 2019

